# Neuroprotective Properties of Dimethyl Fumarate Measured by Optical Coherence Tomography in Non-inflammatory Animal Models

**DOI:** 10.3389/fneur.2020.601628

**Published:** 2021-01-13

**Authors:** Michael Dietrich, Christina Hecker, Milad Nasiri, Sogol Samsam, Andrea Issberner, Zippora Kohne, Hans-Peter Hartung, Philipp Albrecht

**Affiliations:** Department of Neurology, Medical Faculty, Heinrich-Heine University Düsseldorf, Düsseldorf, Germany

**Keywords:** dimethyl fumarate, neuroprotection, optical coherence tomography, optic nerve crush, light-induced photoreceptor loss

## Abstract

While great advances have been made in the immunomodulatory treatment of multiple sclerosis (MS), there is still an unmet need for drugs with neuroprotective potential. Dimethyl fumarate (DMF) has been suggested to exert both immunomodulatory and neuroprotective effects in MS. To investigate if DMF has neuroprotective effects independent of immunomodulation we evaluated its effects in the non-inflammatory animal models of light-induced photoreceptor loss and optic nerve crush. This might also reveal applications for DMF besides MS, such as age related macular degeneration. Retinal neurodegeneration was longitudinally assessed by *in vivo* retinal imaging using optical coherence tomography (OCT), and glutathione (GSH) measurements as well as histological investigations were performed to clarify the mode of action. For light-induced photoreceptor loss, one eye of C57BL/6J mice was irradiated with a LED cold light lamp while for optic nerve crush the optic nerve was clamped behind the eye bulb. The other eye served as control. GSH was measured in the optic nerve, choroid and retina and immunohistological staining of retinal microglia (Iba1) was performed. Mice were treated with 15 or 30 mg DMF/kg bodyweight or vehicle. While no protective effects were observed in optic nerve crush, in the light-induced retinal degeneration model DMF treatment significantly reduced retinal degeneration. In these mice, GSH levels in the retina and surrounding choroid were increased and histological investigations revealed less microglial activation in the outer retinal layers, suggesting both antioxidant and anti-inflammatory effects.

## Introduction

There is an urgent unmet need for new therapeutic approaches effectively preventing the chronic progression of disability and promoting repair in autoimmune diseases of the central nervous system like multiple sclerosis (MS) (1). Fumaric acid esters including dimethyl fumarate (DMF) have previously been used to treat autoimmune disorders like psoriasis and arthritis, where they exert anti-inflammatory effects (2). After two positive phase II trials (3, 4), DMF proved positive for most primary and secondary outcome parameters in the subsequent two large phase-III-trials (5, 6) and has been approved as a disease-modifying therapy for the treatment of relapsing MS. The immunomodulatory effects of DMF have been studied *in vitro* and *in vivo*, revealing effects in several cell types, in particular T-cells. DMF and other fumaric esters have been reported to induce a shift from “Th1” cytokines (IL-2, TNF-α, IFN-γ) to “Th2” cytokines (IL-4, IL-5) as part of their treatment effect in human psoriasis. On the molecular level, these effects were reported to be due to inhibition of the NF-κB pathway (7).

*In vitro*, DMF and its primary metabolite monomethyl fumarate stabilized Nuclear factor erythroid 2-related factor 2 (Nrf2) and stimulated the Nrf2-dependent transcriptional activity of genes with antioxidant response elements (ARE) in their promoters, thereby increasing the expression of the ARE-driven genes NQO1, xCT, and GCL (8–10). *In vivo*, increased levels of Nrf2 and NQO1 activity were detected in the CNS of DMF-treated animals (9, 10). In MS patients, DMF treatment affected mainly memory T cells, resulting in a shift from Th1 toward Th2 responses (11). Although these anti-inflammatory and neuroprotective effects are attributed to DMF, its multifactorial mode of action is still not fully unraveled.

Optical coherence tomography (OCT) is a fast, non-invasive, interferometric technique allowing high resolution imaging of the eye's retina in patients and mice (12, 13). The *in vivo* assessments of retinal neurodegeneration hence allow preclinical studies that are directly transferable to clinical trials. Furthermore, the retinal degeneration of MS patients not only represents a morphological correlate of the functional visual deficits but also mirrors the overall disability assessed by clinical scores (14). Therefore, the anterior visual pathway is increasingly being used for clinical trials evaluating neuroprotective or remyelinating strategies (13). This makes OCT an ideal tool for visualizing the potential of DMF to prevent from neuroaxonal degeneration. In this study, we therefore investigated the effect of DMF in the non-inflammatory models optic nerve crush (ONC) and light-induced photoreceptor loss (Li-PRL). Axonal injury is a major pathological event during MS and therefore the ONC is suited to study protective effects of DMF independently of immunomodulation. The light-induced stress model might not be directly related to MS, however, the retinal degeneration is resulting from overstimulation of photoreceptors leading to accumulation of reactive oxygen species, which play a major role in MS. Additionally the model might reveal treatment options for other ocular pathologies, such as age related macular degeneration (AMD).

After the ONC and retinal irradiation an analysis of the visual pathway by OCT, histology and measurement of the important antioxidant glutathione was performed.

## Materials and Methods

### Optic Nerve Crush

For the optic nerve crush, female, 6 weeks old C57BL6/J mice were used. The optic nerve was grasped approximately 3 mm from the globe with a bended forceps for 10 s. To assure a standardized clamping pressure only the self-clamping mechanism of the forceps was used to crush the nerve. The other eye served as control. DMF stock solution was prepared at 20 mg/mL in dimethyl sulfoxide (DMSO, Sigma-Aldrich) and stored at −80°C until use. Treatment started 1 week before the surgery by adding DMF for verum therapy or DMSO alone for vehicle control to the drinking water. Drinking water was replaced twice a week, uptake was measured daily, and the concentrations of the substances were adjusted to a daily treatment dose of 15 and 30 mg/kg body weight (BW) DMF per day.

### Light-Induced Photoreceptor Loss

Mice (female, 6 weeks old C57BL6/J) were anesthetized (oxygen 20 mL/min with isoflurane (2 vol.%)) and the pupil of the eye was dilated with 0.5% Tropicamide/2.5% Phenylephrine before irradiation. An eye gel was applied to both eyes to prevent dehydration and the formation of cataracts. One eye was irradiated with an LED cold light lamp (KL 1500 LCD, Carl Zeiss AG, Germany) for 10 min at maximum light intensity and fully opened shutter (600 lumen, distance of optical fiber head and eye: 3 cm). The other eye was covered and served as control. During the irradiation procedure, the animals were kept warm by a heating mat. DMF treatment was started 1 week before the irradiation with 30 mg/kg BW or vehicle per os.

### Optical Coherence Tomography Measurement

**Figure 1:**
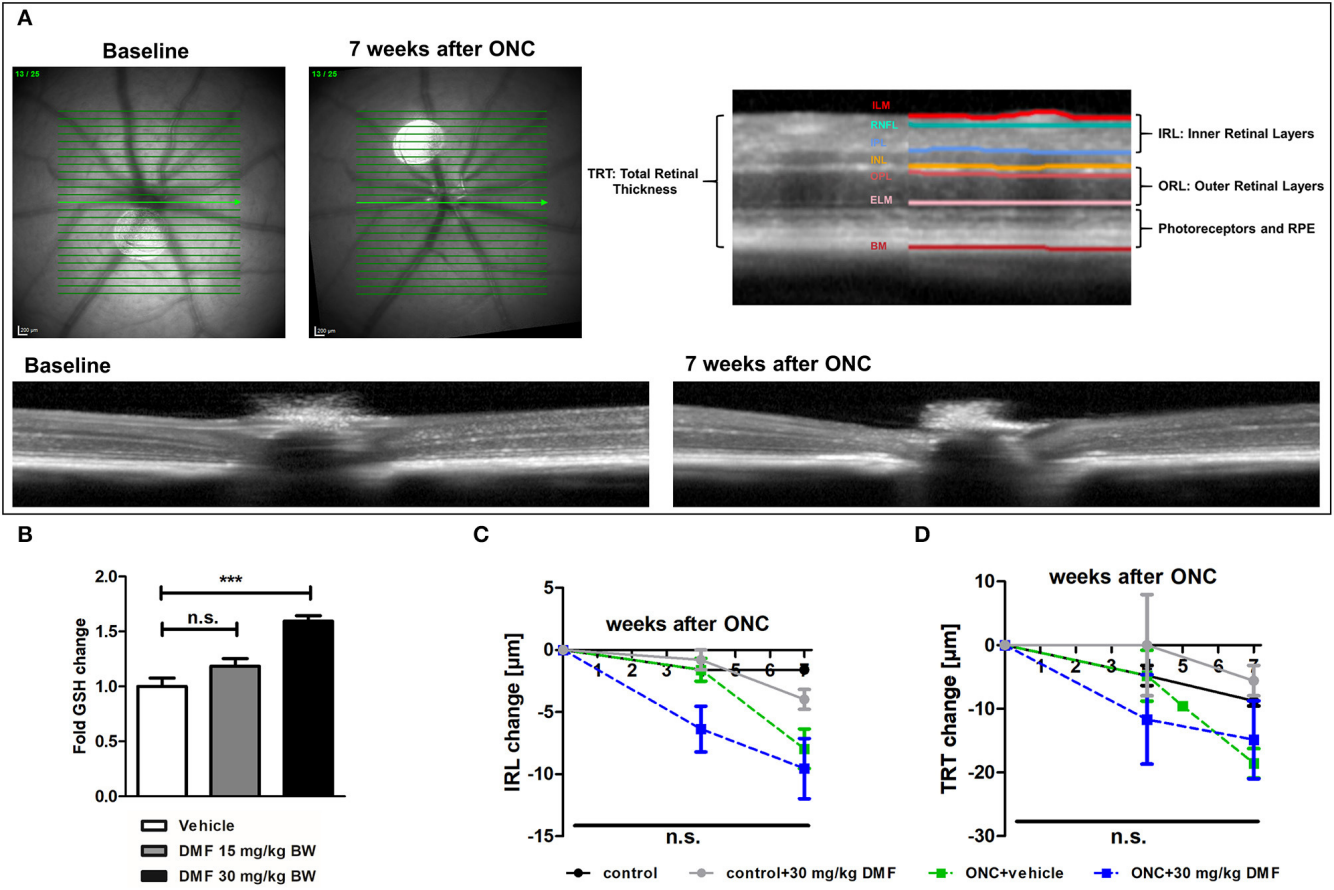
OCT scan of C57Bl/6J mouse retina and ON-crush with GSH in optic nerve and retinal scans. **(A)** Retinal fundus and B-scans of mice before and 7 weeks after ON-crush with Heidelberg Engineering Spectralis™ HRA+OCT device with semi-automated segmentation of the retinal layers. ILM, Inner Limiting Membrane; RNFL, Retinal Nerve Fiber Layer; IPL, Inner Plexiform Layer; INL, Inner Nuclear Layer; OPL, Outer Plexiform Layer; ELM, External Limiting Membrane; BM, Bruch's Membrane; RPE, Retinal Pigment Epithelium. **(B)** The GSH concentration in optic nerve tissue of mice treated with 15 or 30 mg/kg BW DMF at 7 weeks after ONC. Degeneration of the **(C)** total retinal thickness and **(D)** inner retinal layers and of mice over 7 weeks after ONC. All graphs represent the pooled mean ± SEM; *n* = 3 animals per group. ***p < 0.001 by ANOVA with Dunnett's *post hoc* test compared to vehicle for **(A)** and area under the curve compared by ANOVA with Dunnett's *post hoc* test for time courses compared to control for **(C)** and **(D)**.

The measurements of retinal layers were performed using a Spectralis™ HRA+OCT device (Heidelberg Engineering, Germany) under ambient light conditions. The OCT device was equipped with several adaptions for rodents described elsewhere (15) and the scanning protocol was executed as previously described (16). Volume scans were used, which have recently reported to provide excellent inter-rater reliability (interclass correlation coefficient above 0.9) and high reproducibility (17). All measurements were performed using the TruTrack^TM^ automated eye-tracking system incorporated in the Heidelberg Eye Explorer™ software, ensuring that the same area of the retina was assessed in all follow-up measurements. We report the methodology in line with the APOSTEL recommendations (18). Automated segmentation was carried out by the Heidelberg Eye Explorer™ software version 1.9.10.0 followed by manual correction of an investigator, blinded for the experimental groups. We calculated the total thickness of the retina (TRT), the inner retinal layers (IRL), consisting of the retinal nerve fiber layer, ganglion cell layer and inner plexiform layer as described elsewhere (19) as well as the outer retinal layers (ORL), consisting of the outer plexiform layer, outer nuclear layer and the photoreceptors. The different layers of the OCT scans are illustrated in Figure 1A. High-resolution mode was used; only scans with a quality of at least 20 decibels were included.

### Iba1 Staining of the Optic Nerve

At the endpoint of the experiment (7 weeks for ONC and 10 weeks for Li-PRL), mice were sacrificed with an overdose of Isofluran (Piramal Critical Care). Ketamine (50 mg/kg, i.p.) was administered for analgesia before cardiac perfusion was performed with cold phosphate-buffered saline (PBS). Eyes were isolated and fixated in 4% PFA over night at 4°C and dehydrated in ethanol solutions with increasing concentrations. After embedding in paraffin (Paraplast, Leica, Germany), longitudinal sections of 5 μm were cut for immunohistological analysis. Slices of the retinae were incubated with an Iba1 antibody (1:500, Wako chemicals). Cy3 anti-rat (1:500, Millipore) was used as secondary antibody. Microglial infiltration and activation was quantified by fluorescence intensity measurement of the Iba1 staining. Fluorescence stained longitudinal optic nerve sections were acquired with a Leica HyD detector attached to a Leica DMi8 confocal microscope (63x objective lens magnification). At least four sections of the optic nerve from one eye of each mouse were analyzed per staining.

### Glutathione Measurement

For glutathione (GSH) measurements, frozen tissue samples (optic nerve, choroid or retina) from the endpoint of the experiment were homogenized using a micro pestle in PBS/EDTA buffer, sonicated and transferred to lysis buffer. Tissue was further processed and measured enzymatically as previously described (8) using the whole protein amount assessed by the bicinchoninic acid assay for normalization.

### Ethics

All animal procedures were performed in compliance with the experimental guidelines approved by the regional authorities (The Ministry for Environment, Agriculture, Conservation and Consumer Protection of the State of North Rhine-Westphalia; AZ 84-02.4.2014.A059 and AZ 84-02.04.2016.A137) and conform to the Association for Research in Vision and Ophthalmology (ARVO) Statement for the Use of Animals in Ophthalmic and Vision Research.

### Statistics

Statistical analysis was performed using Prism 5 (version 5.00, Graphpad Software, Inc., USA) and IBM SPSS Statistics (version 20, IBM Corporation, USA). A one-way analysis of variance (ANOVA) with Dunnett's *post hoc* test was used to compare means of multiple groups to the control group and a Student's *t*-test was used to compare the means of two groups for histology and GSH measurements. For these analyses, one eye/optic nerve per animal was included in the analysis. For *in vivo* measurements, differences in retinal thickness were analyzed using area under the curve compared by ANOVA followed by Dunnett's *post hoc* test.

## Results

### DMF Is Not Effective in Optic Nerve Crush

As first experimental approach, an optic nerve crush was performed in C57Bl/6J mice with and without prophylactic DMF (15 or 30 mg/kg BW, per os) treatment, starting 1 week before the surgery. Treatment with 30 mg/kg BW, but not 15 mg/kg BW DMF resulted in an increased GSH level in the optic nerve 7 weeks after the crush (Figure 1B). The retinal thickness was measured by OCT over 7 weeks with the follow-up function of the Heidelberg Engineering software. The measurement revealed that the optic nerve crush led to a strong degeneration of the inner retinal layer (IRL) (Figures 1A,C) and a decrease of the total retinal thickness. The loss of retinal tissue was a dynamic process, showing continuous degeneration over the 7 weeks (Figure 1D). We found an elevated level of the antioxidant GSH in the optic nerve of mice treated with 30 mg/kg BW DMF, while 15 mg/kg did not significantly increase GSH. The augmented GSH level was however not accompanied by a protective effect of DMF from tissue loss: Retinal degeneration after ONC progressed at the same level under DMF therapy as in vehicle treated mice (Figures 1C,D). Of note, the contralateral eye, which served as an internal control (no crush), also showed retinal degeneration at 7 weeks after the crush (TRT: −7.16 μm ± 2.4 μm; IRL: −2.78 ± 1.3 μm).

### DMF Reveals Protective Capacities After Light-Induced Retinal Damage

**Figure 2:**
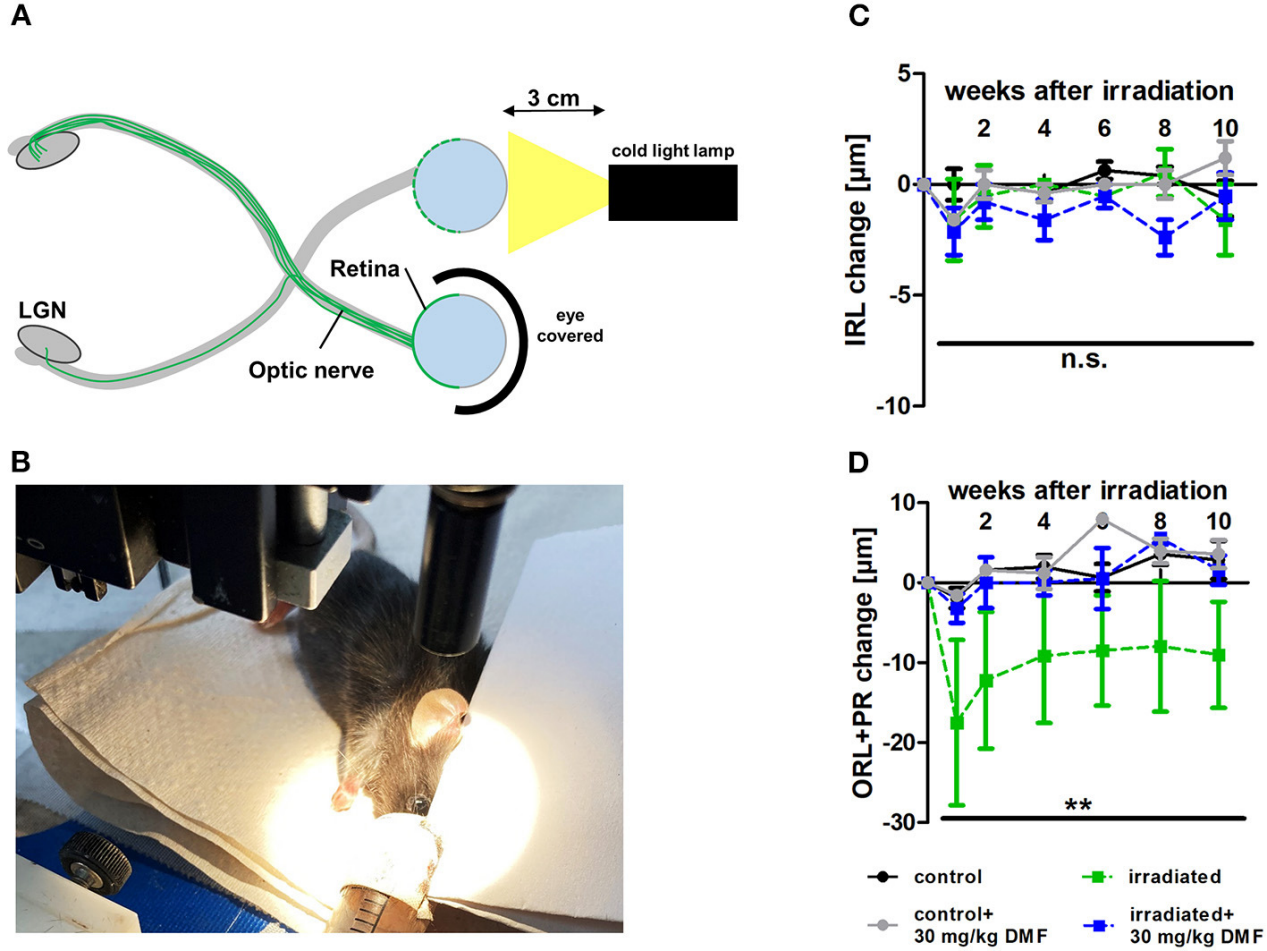
Retinal degeneration after light-induced photoreceptor loss (Li-PRL). **(A)** Schematic image of Li-PRL and **(B)** image of irradiation of C57Bl/6J mouse. **(C)** Change of inner retinal layers (IRL) and **(D)** outer retinal layers (ORL) with photoreceptors (PR) of mice over 10 weeks after Li-PRL. All graphs represent the pooled mean ± SEM; *n* = 3 animals per group. **p < 0.01 by area under the curve compared by ANOVA with Dunnett's *post hoc* test compared to irradiated control. LGN = lateral geniculate nucleus.

As DMF led to an increase of total glutathione in optic nerve tissue in the ONC model, suggesting possible protective effects in a less severe model, light-induced photoreceptor loss was performed, treating animals using the same dosing protocol: DMF 30 mg/kg BW, per os, starting 7 days before the irradiation. One eye was irradiated at maximum power (600 lumen) for 10 min, while the other eye was covered and served as a control (Figures 2A,B). While the inner retinal layer thickness remained unchanged after irradiation over 10 weeks regardless of the treatment (Figure 2C), the outer retinal layers and photoreceptors showed a strong degeneration already after 1 week, regaining thickness until week 4 after Li-PRL. In contrast to the ONC, in mice treated prophylactically with 30 mg/kg BW DMF, the degeneration of the ORL and photoreceptors was completely prevented (Figure 2D) over 10 weeks.

### Protection by DMF Is Mediated by Anti-inflammatory and Antioxidant Properties

**Figure 3:**
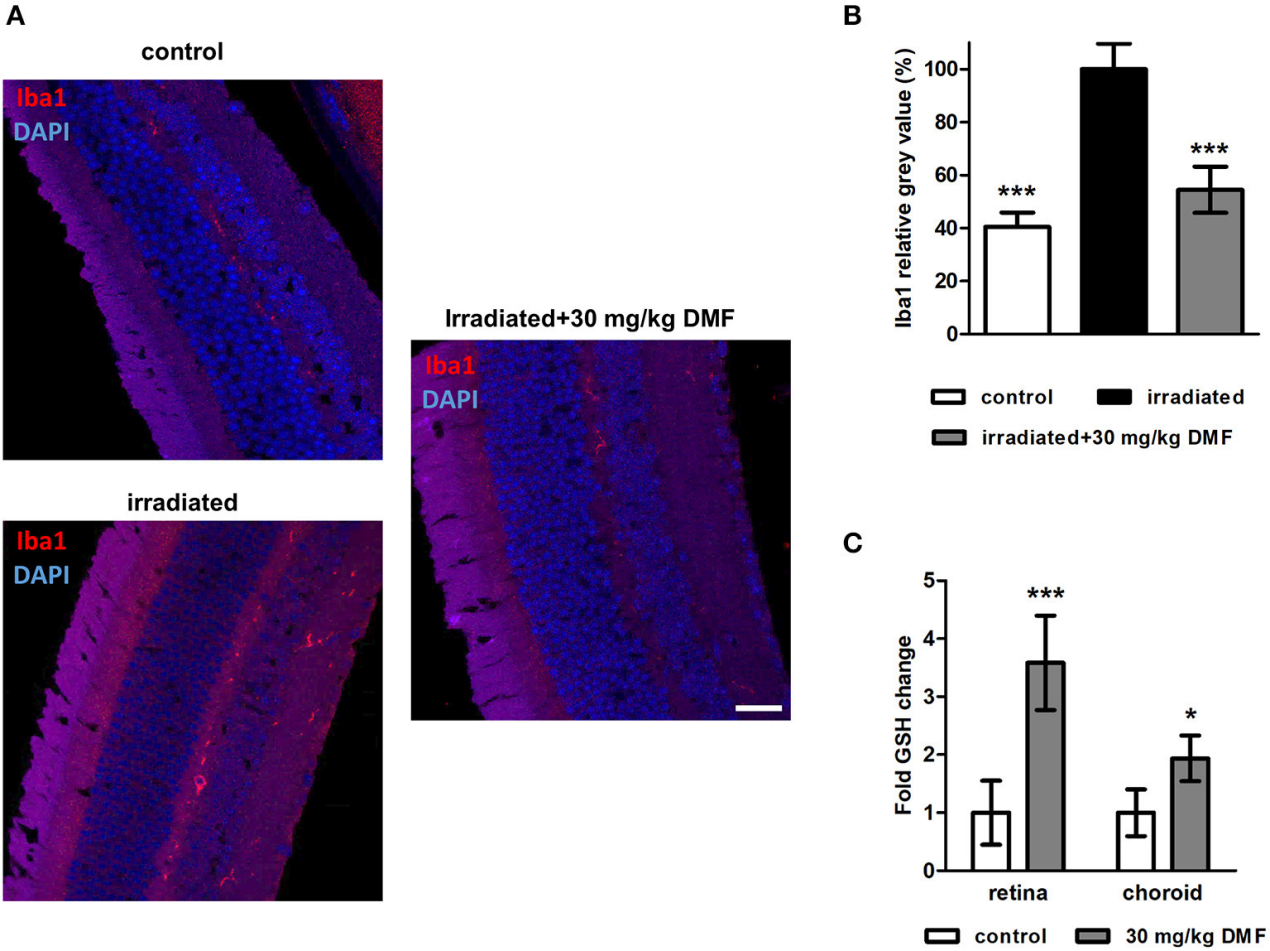
Microglia staining and GSH measure in visual system after Li-PRL. **(A)** Longitudinal sections of retinae of mice 10 weeks after Li-PRL stained for Iba1. **(B)** Quantitative analyses of microglial activation by fluorescence intensity measurement; one eye per mouse was included. **(C)** GSH concentration in retina and choroid tissue of mice treated with 30 mg/kg BW DMF. All graphs represent the pooled mean ± SEM; *n* = 3 animals per group. ***p < 0.001, *p < 0.05 by ANOVA with Dunnett's *post hoc* test.

Ten weeks after the Li-PRL, mice were sacrificed and longitudinal sections of the retina were stained for Iba1, a protein highly expressed in activated microglia and macrophages (Figure 3A). The irradiation led to an increased of Iba1^+^ cells, suggesting an enhanced microglial activation and/or macrophage infiltration, even 10 weeks after the overstimulation of the photoreceptors. These cells were mainly located in the outer retinal layers. In the non-irradiated control eye, almost no Iba1^+^ cells were detectable. DMF treatment diminished the increased activation of microglia and macrophages, leading to a baseline level of Iba1 in the retina (Figure 3B). Enzymatic measurement of the antioxidant GSH in the retina and surrounding choroid tissue of DMF treated mice showed an upregulation of total tissue glutathione by 3.5- and 2-fold, respectively (Figure 3C). The irradiation itself had no effect on the GSH level in the tissue (data not shown).

## Discussion

In the experimental autoimmune encephalomyelitis and optic neuritis (EAEON) model, an animal model for MS, DMF has already been extensively tested (9, 20, 21). This inflammatory model, inducing prominent retinal degeneration (17), is frequently used to study the protective effects of therapeutics (13). However, an increasing body of evidence suggested effects of DMF beyond the immunomodulatory characteristics (8, 9, 22). We therefore sought to characterize the effects of DMF independently of its immunomodulatory capacities using mouse models of non-inflammatory axonal damage and retinal degeneration. While the optic nerve crush induces axonal injury, which is also occurring in the progression of optic neuritis and MS, light-induced retinal stress might as well-model other ocular pathologies, such as AMD (23). In AMD, one of the most common vision-threatening diseases, the RPE is physiologically exposed to high levels of oxidative stress during its lifespan. For the well-functioning of its antioxidant systems, the Nrf2-pathway plays an important role (24). Both approaches led to a prominent degradation of retinal tissue. While the ONC damages the optic nerve mainly inducing a degeneration of the inner retinal layers, the light-induced damage affects the cells in the outer retinal layers including the photoreceptors. While the axonal damage of the optic nerve in the ONC model results in indirect Wallerian degeneration of the RNFL, the ganglion cells and their dendritic arbor, the light induced injury directly damages the retinal pigment epithelium (RPE), the photoreceptors and their cell bodies and synaptic processes in the outer nuclear and outer plexiform layer, respectively. Of note, we also observed retinal degeneration after ON-crush in the contralateral, untreated eye. This might be due to sympathetic ophthalmia, which is a serious, bilateral uveitis that occurs after either eye surgery or penetrating or perforating eye trauma (25). The traumatic injury on the optic nerve and the surrounding tissue might therefore lead to impairment of the contralateral nerve and eye. Numerous other studies in rodents investigating retinal degeneration after ONC also found a decrease of the RNFL and TRT, as well as a loss of retinal ganglion cells (26–29). After a light-induced retinal damage, authors essentially found degeneration of the ORL and photoreceptors, in line with our results (30, 31).

Interestingly, DMF treatment only prevented retinal degeneration after Li-PRL, but not after ONC. The axonal damage in the ONC model is apparently too severe to be susceptible to the protective effects of DMF despite evidence of GSH increase in retinal tissue. Possibly, oxidative stress may not play an important role for the apoptosis of retinal ganglion cells (RGCs) in the context of the Wallerian degeneration resulting from ONC. On the other hand, the Li-PRL is resulting from overstimulation of photoreceptors leading to accumulation of reactive oxygen species, calcium overload and finally degeneration of the photoreceptors and their neurons in the outer nuclear layer. This degeneration is tightly linked to oxidative stress, which is more likely to be sensitive to the antioxidant mode of action of DMF and the resulting GSH increase. Other studies also found an enhancement of antioxidant pathways after DMF therapy. Treatment of mice with DMF or MMF resulted in increased nuclear levels of active Nrf2, with subsequent up-regulation of canonical antioxidant target genes with the effect being lost in mice lacking Nrf2 (10). In another study, DMF increased immunoreactivity for Nrf2 in neurons of the motor cortex and the brainstem as well as in oligodendrocytes and astrocyte in experimental autoimmune encephalomyelitis (EAE) mice (9). Contrastingly, Schulze-Topphoff and colleagues reported, that oral DMF therapy protected wildtype and Nrf2 deficient mice equally well from development of clinical and histologic EAE, suggesting, that the anti-inflammatory activity of DMF may occur through alternative pathways (21). *In vitro*, DMF and MMF significantly improved cell viability of astrocytes or neurons and increased glutathione levels after toxic oxidative challenge in a concentration-dependent manner (10). Additionally, DMF treatment stabilized Nrf2 and stimulated the Nrf2-dependent transcriptional activity of genes with ARE in their promoters (8, 9). In another *in vitro* study by our lab, DMF was demonstrated to increase GSH recycling through induction of glutathione reductase in the hippocampal neuronal cell line HT22 (22). Similar to our study, but in another mouse line, Jiang and colleagues treated albino BALB/c mice intraperitoneally with monomethyl fumarate (MMF), the primary metabolite of DMF, before light exposure for 1 h. 7 days later, mice were examined by OCT. MMF treatment prevented morphologic changes in the ORLs and photoreceptor layers in a dose-dependent manner (32). In an *in vitro* study, Nrf2 protected mouse photoreceptor cells from photo-oxidative stress induced by blue light (33). Interestingly, in the MS brain Nrf2 expression varies in different cell types and is associated with active demyelination in the lesions. Nuclear Nrf2 expression was particularly observed in oligodendrocytes, while only a minor number of Nrf2-positive neurons were detected, even in highly inflammatory cortical lesions. In degenerating cells, which showed signs of apoptotic or necrotic cell death, the most prominent Nrf2 expression was found (34).

To analyze the anti-inflammatory mode of action of DMF, we stained longitudinal retinal sections with the microglia marker Iba1. We found, that after light exposure, microglial activation was prominently increased in the ORL. In mice treated with DMF, this effect was diminished showing almost no Iba1 positive cells, similar to the non-irradiated condition. Similar findings were reported in the study of Jiang et al., where expression of the microglial marker Cd14 was upregulated after light exposure, but suppressed after MMF treatment (32). In an *in vivo* EAE study, treatment with DMF ameliorated clinical disability in C57Bl/6 mice and modulated activated microglia from a classically activated, pro-inflammatory phenotype to an alternatively activated, neuroprotective phenotype, presumably by activation of the hydroxycarboxylic acid receptor 2 (35).

Our own findings of neuroprotective capacities of DMF in the non-inflammatory model of light induced photoreceptor loss corroborate and extend previous reports and suggest that both antioxidant mechanisms leading to elevated GSH levels and immunomodulatory effects reducing microglial activation are involved. A limitation of the study is that the Li-PRL does not have a direct link to MS. We also do not show positive effects of DMF in an inflammatory model, which have however already been well-documented in numerous EAE and MS studies (5, 6, 8, 9, 20, 21). The main goal of our study was, to explore the effects of DMF beyond immunomodulation. After irradiation of the retina, the degeneration of the photoreceptors and outer retinal layers is mainly caused by the accumulation of reactive oxygen species, which is not only a pathological hallmark in MS, but also of other eye-related disorders. The fact that we observed no protective effects in the ONC model suggests that, despite upregulation of GSH, DMF may not be effective in traumatic axonal damage.

## Data Availability Statement

The original contributions generated for this study are included in the article/supplementary material, further inquiries can be directed to the corresponding author/s.

## Ethics Statement

The animal study was reviewed and approved by The Ministry for Environment, Agriculture, Conservation and Consumer Protection of the State of North Rhine-Westphalia: AZ 84-02.4.2014.A059 and AZ 84-02.04.2016.A137.

## Author Contributions

MD, CH, MN, SS, AI, and ZK performed the experiments and analyzed the data. MD and PA wrote the manuscript. H-PH was involved in revising the manuscript critically for important intellectual content and made substantial contributions to interpretation of data. PA and MD conceived the study and supervised experiments. All authors read and approved the final manuscript.

## Conflict of Interest

The authors declare that they have no conflict of interest related to the work presented. The following financial disclosures are unrelated to the work MD received speaker honoraria from Novartis and Merck. H-PH has received fees for serving on steering and data monitoring committees from Bayer Healthcare, Biogen, Celgene BMS, CSL Behring, GeNeuro, MedImmune, Merck, Novartis, Octapharma, Roche, Sanofi Genzyme, TG Therapeutic sand Viela Bio; fees for serving on advisory boards from Biogen, Sanofi Genzyme, Merck, Novartis, Octapharma, and Roche; and lecture fees from Biogen, Celgene BMS, Merck, Novartis, Roche, Sanofi Genzyme. PA received compensation for serving on Scientific Advisory Boards for Ipsen, Novartis, Biogen; he received speaker honoraria and travel support from Novartis, Teva, Biogen, Merz Pharmaceuticals, Ipsen, Allergan, Bayer Healthcare, Esai, UCB and Glaxo Smith Kline; he received research support from Novartis, Biogen, Teva, Merz Pharmaceuticals, Ipsen, and Roche. The remaining author report no disclosures.
